# Sampling and counting genome rearrangement scenarios

**DOI:** 10.1186/1471-2105-16-S14-S6

**Published:** 2015-10-02

**Authors:** István Miklós, Heather Smith

**Affiliations:** 1 MTA Rényi Institute, Reáltanoda u. 13-15, 1053 Budapest, Hungary; 2 MTA SZTAKI, Lágymányosi u. 11, 1111 Budapest, Hungary; 3 University of South Carolina, 1523 Greene Street, Columbia, SC, 29208 USA

**Keywords:** Genome rearrangement, computational complexity, Gibbs sampling, Single Cut or Join

## Abstract

**Background:**

Even for moderate size inputs, there are a tremendous number of optimal rearrangement scenarios, regardless what the model is and which specific question is to be answered. Therefore giving one optimal solution might be misleading and cannot be used for statistical inferring. Statistically well funded methods are necessary to sample uniformly from the solution space and then a small number of samples are sufficient for statistical inferring.

**Contribution:**

In this paper, we give a mini-review about the state-of-the-art of sampling and counting rearrangement scenarios, focusing on the reversal, DCJ and SCJ models. Above that, we also give a Gibbs sampler for sampling most parsimonious labeling of evolutionary trees under the SCJ model. The method has been implemented and tested on real life data. The software package together with example data can be downloaded from http://www.renyi.hu/~miklosi/SCJ-Gibbs/

## Background

The minimum number of mutations necessary to transform one genome into another is only one of the statistics that describe the evolutionary relationship between genomes. By definition, this number is constant for any most parsimonious rearrangement scenario. On the other hand, other statistics like the breakpoint reuse [1,2], sizes and positions of inversions [3,4] vary among the possible solutions, and drawing these values from a single optimal solution might be statistically biased. Instead of highlighting one most parsimonious solution, we are interested in expectations of the above mentioned statistics, for example, what is the expected usage of a particular breakpoint, what is the expected (average) size of reversals. Statistical samples are needed for hypothesis testing, too, like testing the Random Breakpoint Model [1,2] or the hypothesis that there is selection for maintaining balanced replichors [4].

The final goal is to sample rearrangement scenarios from a statistically well-funded distribution, for example, from some Bayesian distribution. Some efforts have been made to develop Monte Carlo methods to sample from such distributions [5-7].

The theoretical study of the computational efficiency of the Monte Carlo methods is in its childhood, and the first attempts use simplifications. A possible simplification is to restrict the distribution only for the most parsimonious solutions. This restricted distribution will be the uniform one when the rearrangement model is the reversal model [8] and will be close to the uniform distribution in case of the DCJ and SCJ models [9]. It is well known that sampling from a distribution close to the uniform distribution and sampling from the uniform distribution have similar computational complexity, since importance sampling or rejection sampling can be used to transform one of the problems into the another [10]. Therefore studying the computational complexity of sampling from the uniform distribution is theoretically well-funded even for the DCJ and SCJ models.

The general theory of the computational complexity of counting combinatorial objects as well as sampling from the uniform distribution of them has been developed since the late seventies and eighties [11,12]. In this paper, we give an overview of what we know about sampling and counting genome rearrangement scenarios, what are the proved theorems and what are the conjectures and open questions. Above that, we give a Gibbs sampler for sampling uniformly the most parsimonious labeling of internal nodes of a rooted binary tree under the SCJ model. This sampling problem has an unknown computational complexity, and as a first step towards resolving its computational complexity, we prove the irreducibility of the Gibbs sampler. The method has been tested on real life data.

### Complexity classes

Below we review the complexity classes needed in this paper together with the important main theorems. First we start with the decision problems since the counting problems are defined via them and also main theorems on counting complexity classes which use these decision complexity classes.

**Definition 1 ***A decision problem is in *P *if a deterministic Turing machine can solve it in polynomial time. This means that there are efficient algorithms that can quickly solve such problems*.

*A decision problem is in *NP *if a non-deterministic Turing machine can solve it in polynomial time. An equivalent definition is that a witness, namely, a solution that proves the "yes" answer to the question, can be verified in polynomial time*.

*Finally, a decision problem is in *NP-complete *if it is in *NP *and any problem in *NP *is polynomial reducible to it. Formally, optimization problems as hard as the *NP-complete *problems are called *NP-hard *problems*.

Polynomial reducibility means that any problem in NP could be solved efficiently if any NP-complete problem was solvable efficiently.

To give examples from the field of genome rearrangements, deciding if *k *number of reversals is sufficient to transform a genome into another is in P [13], while deciding if *k *number of reversals is sufficient to explain the evolutionary history of three genomes is NP-complete and finding the evolutionary history with the minimum number of reversals is NP-hard. [14].

**Definition 2 ***A decision problem is in *RP *if a random algorithm exists with the following properties: a) the running time is deterministic and grows polynomially with the size of the input, b) if the answer is "no," then the algorithm answers "no" with probability 1, c) if the answer is "yes," then it answers "yes" with probability at least 1/2*.

We know that P ⊆ RP ⊆ NP. In this paper, we will assume that RP ≠ NP and thus P ≠ NP.

Now we turn to define counting problems.

**Definition 3 ***A counting problem is in *#P *if it asks for the number of witnesses of an *NP *problem*.

*A counting problem in *#P *is in *FP *if it can be solved in polynomial time*.

*A counting problem in *#P *is in *#P-complete *if any problem in *#P *can be reduced to it by a polynomial time counting reduction*.

Hard decision problems cannot be counted easily. Although if a decision problem *X *is in NP-complete, it does not necessarily imply that the corresponding counting problem #*X *is in #P-complete, however, it is easy to see if #*X *was in FP that would immediately imply that P = NP. To highlight this fact with a genome rearrangement problem, let *X *be the decision problem if *k *reversals are sufficient to explain the evolutionary history of three genomes, and let #*X *be the counting problem how many evolutionary history of three genomes exist with *k *reversals. If there was an efficient algorithm to count the later, the decision problem would be also easy: when the number of evolutionary histories is 0 for a particular *k *and three genomes, the answer would be "no" for the question whether or not such history exists.

If a decision problem is easy, the corresponding counting problem might still be hard. In his seminal paper in which the #P complexity class has been defined, Valiant proved that counting the number of perfect matchings in a bipartite graph is #P-complete, although finding one perfect matching is easy [11].

Counting problems also have random approximation algorithms. The two main complexity classes are the following.

**Definition 4 ***A counting problem in *#P *is in *FPRAS *(Fully Polynomial Randomized Approximation Scheme) if there exists a randomized algorithm such that for any instance x, and ϵ, δ *> 0, *it generates an approximation *$\hat{f}$*for the solution f, satisfying*

(1) $$P\left(\frac{f}{1+\epsilon }\leq \hat{f}\leq f(1+\epsilon )\right)\geq \text{1}-\delta ,$$

*and the algorithm has a running time bounded by a polynomial of *|*x*|, 1/*ϵ*, − log(*δ*). *Such an algorithm is also called an *FPRAS *algorithm and we will also say equivalenty that the problem has an *FPRAS *approximation*.

**Definition 5 ***A counting problem in *#P *is in *FPAUS *(Fully Polynomial Almost Uniform Sampler) if there exists a randomized algorithm such that, for any instance x and ϵ *> 0, *it generates a random element of the solution space (the set of solutions) following a distribution p satisfying*

(2) $$d_{TV}\left(U,p\right)\leq \epsilon$$

*where U is the uniform distribution over the solution space, and the algorithm has a time complexity bounded by a polynomial of |x|, and − *log(*ϵ*). *Here d_TV _denotes the total variation distance between two probaility distributions over the same finite domain, by definition, the total variation distance of p and π is*

(3) $$d_{TV}\left(p,\pi \right):=\frac{1}{2}\underset{x\in X}{\sum }|p\left(x\right)-\pi \left(x\right)|$$

*Such an algorithm is also called *FPAUS *and we will also say that a problem has an *FPAUS.

The two counting classes have a strong correspondence. Jerrum, Valiant and Vazirani proved that any counting problem belonging to a large class of counting problems is in FPRAS if and only if it is in FPAUS [12]. The proof is constructive, so given one of the algorithms, the other one can be explicitly constructed. This large class is called self-reducible counting problems. Here we skip the formal definition. Informally, a self-reducible counting problem is such that the extension of any prefix of a partial solution is the solution of another problem instance (and other mild technical conditions are necessary, the exact definition can be found in [12]). For example, any genome rearrangement problem asking for most parsimonious genome rearrangement scenarios are self-reducible counting problems. Indeed, assume that a genome *G*_1 _has started transforming into *G*_2 _in a most parsimonious way. If a few transformations *tr *are applied on *G*_1_, then the possible finishing of this partial scenario are the most parsimonious rearrangement scenarios between *G*_1 _∗ *tr *and *G*_2_, where *G*_1 _∗ *tr *denotes the genome we get by applying the transformations *tr *on *G*_1_. For self-reducible counting problems, FPRAS algorithms are frequently given via FPAUS, and FPAUS is given via rapidly mixing Markov chains. We skip the definition of rapidly mixing Markov chains. Roughly speaking, a Markov chain is rapidly mixing if it can be used for an FPAUS algorithm.

It is hard to count, even approximately, the number of witnesses of a hard decision problem. It is easy to see that an FPRAS algorithm for a #*X *counting problem whose corresponding decision problem *X *is in NP-complete would imply that RP = NP [15].

Even easy decision problems might be hard to count approximately. Jerrum, Valiant and Vazirani proved that an FPRAS algorithm for counting the number of cycles in a directed graph would imply that RP = NP [12].

On the other hand, there are #P-complete problems that have FPRAS approximations. An example for this is counting the number of total orderings of partially ordered sets, which has an FPAUS algorithm via a rapidly mixing Markov chain [16] and thus, the problem is also in FPRAS since it is self-reducible. On the other hand, it is #P-complete [17].

To summarize, hard decision problems are hard to count both exactly and approximately, assuming that P ≠ NP and RP ≠ NP. The corresponding counting problem of an easy decision problem might be i) easy (in FP), ii) hard to exactly count (#P-complete) but have a good stochastic approximation (FPRAS) or iii) hard to count even approximately (not in FPRAS assuming that RP ≠ NP). Although no strict trichotomy exists, the majority of the counting problems fall into these three categories just like the majority of the decision problems are either in P or in NP-complete.

The interested reader is referred to the survey book by Mark Jerrum [15], which gives a detailed introduction on the algorithmics of sampling and counting.

### Genome Rearrangement models

Here we consider 3 genome rearrangement models: the reversal, the DCJ and the SCJ model.

#### The reversal model

In the reversal model, genomes are represented as signed permutations. Each number represents a synteny block in a linear, unichromosomal genome. A reversal flips a consecutive part of the permutation, it reverses the order of the number and changes all signs. For example, a reversal from +3 till +5 on permutation (+2 +3 −1 −4 +6 +5 −8 +7) creates permutation (+2 −5 −6 +4 +1 −3 −8 +7). Polynomial running time algorithms exist to calculate the minimum number of reversals transforming a signed permutation into another [13,18,19]. Such series of reversals are called most parsimonious reversal scenarios.

#### The DCJ model

In the Double Cut and Join model, genomes are edge-labeled directed graphs, each label is unique, and each vertex has a total degree (sum of incoming and outgoing edges) either 1 or 2. Such graphs can be uniquely decomposed into paths and cycles. Degree 2 vertices are called adjacencies, degree 1 vertices are called telomeres. Each edge represents a synteny block, thus in this model, genomes are mixed multichro- mosomal genomes, namely, the chromosomes may be both linear and circular. The ends of the edges are called extremities. Since the edges are directed, the two ends are distinguishable. A Double Cut and Join operation takes at most two vertices and shuffles them into at most two new vertices meanwhile keeping the labels of the edges. Finding a shortest DCJ scenario transforming a genome into another can also be done in polynomial time [20].

#### The SCJ model

In the Single Cut or Join model, genomes are modeled exactly in the same way as in the DCJ model. A Single Cut or Join operation either takes an adjacency and cuts it into two parts or takes two telomeres and joins them into an adjacency. In the SCJ model, the simplified representation of the genomes, which is the list of adjacencies that the genome has, is useful. Given a set of common synteny blocks a set of genomes share, each genome can be uniquely represented by its list of adjacencies. With *n *common synteny blocks, $\left(\begin{array}{c} 2n\\ 2 \end{array}\right)$ possible adjacencies can be considered, which have $2^{\left(\begin{array}{c} 2n\\ 2 \end{array}\right)}$ possible subsets. However, not all these subsets represent a genome. We say that two adjacencies are in conflict (or they are conflicting adjacencies) if they share an extremity. It is easy to see that conflict-free sets of adjacencies are exactly the sets of adjacencies that represent genomes [21]. Finding a shortest SCJ scenario is also an easy computational task [21].

## Results

### State-of-the-art of sampling and counting genome rearrangement scenarios

We consider the reversal (REV), DCJ and SCJ models in this section. For each model, five specific questions are considered:

• **Pairwise rearrangement problem **Given two genomes, *G*_1 _and *G*_2_, and one of the rearrangement models, *M *, how many most parsimonious rearrangement scenarios exist that transform *G*_1 _into *G*_2_? We will denote this number by *n_M _*(*G*_1_, *G*_2_) and the counting problem to estimate this number by #M, where M ∈ {REV, DCJ, SCJ}.

• **Most parsimonious median problem **Given a series of genomes, *G*_1_, *G*_2_...*G_k _*, and one of the rearrangement models, *M *, how many genomes *G_m _*exist that minimize $\sum_{i=1}^{k}d_{M}\left(G_{i},\;G_{m}\right)$, where *d_M_*(*G*, *G'*) denotes the minimum number of operations needed to transform *G *into *G' *under the model *M*. We will call each *G_m _*an optimal median. This set will be denoted by $O_{M}(G_{1},G_{2},...G_{k})$.

• **Most parsimonious median scenarios **Given a series of genomes, *G*_1_, *G*_2_...*G_k_*, and one of the rearrangement models, *M *, how many optimal median scenarios exist. That is, count for all optimal medians the number of possible rearrangement scenarios. With a formula, we are looking for

$$\underset{G_{m}\in O_{M}\left(G_{1},G_{2},...G_{k}\right)}{\sum }\overset{k}{\underset{i=1}{\prod }}n_{M}\left(G_{i},\;G_{m}\right).$$

• **Most parsimonious labeling of evolutionary trees **Given one of the rearrangement models, *M *, a rooted binary tree, *T *(*V, E*), where *V *is the disjoint union of leaves *L *and internal nodes *I*. Furthermore, given a function f:L→G that labels the leaves, where G  denotes the set of possible genomes. We are looking for how many functions g:V→G exist such that for any *v *∈ *L*, *g*(*v*) = *f *(*v*) and *g *minimizes

$$\underset{\left(u,v\right)\in E}{\sum }d_{M}\left(g\left(u\right),\;g\left(v\right)\right).$$

We will denote this set of functions by $O_{M}^{\prime }\left(T,\;f\right)$.

• **Most parsimonious scenarios on evolutionary trees **Given one of the rearrangement models, *M *, a rooted binary tree *T *(*V, E*) and a labeling function *f *as described above, we are looking for

$$\;\underset{g\in {O^{\prime }}_{M}\left(T\;,f\right)}{\sum }\underset{\left(u,v\right)\in E}{\prod }n_{M}\left(g\left(u\right),\;g\left(v\right)\right).$$

For each model, we introduce the state-of-the-art of our knowledge. It is also summarized in Table 1.

**Table 1:** The computational complexity of five specific counting problems under three different rearrangement models as described in details in the text.

	Reversal	DCJ	SCJ
Pairwise rearrangement	C: #P-complete	C: #P-complete	T: in FP [30]
	C: in FPRAS	T: in FPRAS [25]	

Median	T: not in FP^‡^	T: not in FP^‡^	T: in FP*
	T: not in FPRAS^‡^	T: not in FPRAS^‡^	

Median scenario	T: not in FP^‡^	T: not in FP^‡^	T: #P-complete[32]
	T: not in FPRAS^‡^	T: not in FPRAS^‡^	U: in/not in FPRAS

Tree labeling	T: not in FP^‡^	T: not in FP^‡^	U: FP/#P-complete
	T: not in FPRAS^‡^	T: not in FPRAS^‡^	U: in/not in FPRAS

Tree scenario	T: not in FP^‡^	T: not in FP^‡^	T: #P-complete[32]
	T: not in FPRAS^‡^	T: not in FPRAS^‡^	T: not in FPRAS [30]

Notations: T: theorem, C: conjecture, U: unknown complexity, and there is no evidence to set up a conjecture favoring one of the possibilities. All theorems are referenced except: ‡: based on the fact that the corresponding optimization problem is NP-hard, *: proved in this paper. In all cases, "not in FP" should be considered under the assumption that P ≠ NP. Similarly, "not in FPRAS" should be considered under the assumption that RP ≠ NP.

#### The reversal model

The reversal model is the computationally most complicated among the three considered models. Finding one optimal median is NP-hard [14], therefore even an FPRAS approximation is not possible for counting the optimal medians assuming that RP ≠ NP. Similarly, counting the optimal medians is not in FP, assuming that P ≠ NP. It is easy to see that finding an optimal median of three genomes is polynomially reducible to finding most parsimonious labelings for evolutionary trees. Indeed, given three genomes *G*_1_, *G*_2 _and *G*_3_, label the leaves of a rooted binary tree with three leaves with *G*_1_, *G*_2 _and *G*_3_, and any most parsimonious labeling of the internal nodes will provide an optimal median: the genome labeling the internal node, which is not the root, is an optimal median. Therefore finding a most parsimonious labeling of evolutionary trees under the reversal model is also NP-hard, and thus, counting the solutions does not admit an FPRAS approximaion assuming that RP ≠ NP and it is not in FP assuming that P ≠ NP. Similarly, any most parsimonious median scenario provides a most parsimonious median, as well as, any most parsimonious scenario on an evolutionary tree provides a most parsimonious labeling, therefore these problems are also NP-hard, and the number of solutions does not have FPRAS approximations assuming that RP ≠ NP and not in FP assuming that P ≠ NP.

The only open question is the complexity of #REV, namely, counting the most parsimonious scenarios between two genomes. No polynomial time algorithm exists for #REV. Siepel [22] developed a method to count all optimal next steps, namely, what are the reversals *ρ *for which

$$d_{\text{REV}}\left(G_{1}\rho ,G_{2}\right)=d_{\text{REV}}\left(G_{1},G_{2}\right)-1,$$

but this cannot give a polynomial time algorithm to calculate the number of most parsimonious sorting scenarios between *G*_1 _and *G*_2_. Since nobody was able to come up with a fast counting algorithm in the last 15 years, #REV is conjectured to be in #P-complete.

Several attempts have been made to develop a rapidly mixing Markov chain converging to the uniform distribution of the most parsimonious scenarios. Such a Markov chain would provide an FPAUS algorithm, and since #REV is self-reducible, this would immediately imply that #REV is in FPRAS. Unfortunately, the only theorem proved here is a negative result: Miklós *et al*. [23] proved that the most commonly used window-resampling Markov chain [5-7] is torpidly mixing. The high level explanation why the window-resampling Markov chain is torpidly mixing is the following. There exist (an infinite series of) genomes *G*_1 _and *G*_2 _having large subsets of most parsimonious rearrangement scenarios *R*_1 _and *R*_2 _such that for any *r*_1 _∈ *R*_1 _and *r*_2 _∈ *R*_2 _scenarios it is impossible to transform *r*_1 _into *r*_2 _by changing only an *o*(|*r*_1_|)[= *o*(|*r*_2_|)] window in each step. With other words, "big jumps" are necessary to move from *R*_1 _to *R*_2_. These big jumps happen to have exponentially small acceptance ratios in the Metropolis-Hastings algorithm for almost all *r*_1 _and *r*_2_, making the Markov chain torpidly mixing.

However, this does not imply that #REV is not in FPRAS, since other methods might lead to rapidly mixing Markov chains. Miklós and Darling [24] and Miklós and Tannier [9] developed parallel Markov chain methods as candidates for rapidly mixing Markov chains for #REV. It is still open whether or not these Markov chains are rapidly mixing.

#### The DCJ model

Finding an optimal DCJ median is also NP-hard, therefore - similar to the reversal model - four of the listed problems do not have an FPRAS approximation assuming that RP ≠ NP. On the other hand, Miklós and Tannier [25] proved that #DCJ is in FPRAS and in FPAUS. They used a Markov chain that walks on subsets of DCJ scenarios and rapidly converges to the distribution proportional to the size of the sets. Each set is such that sharp uniform sampling from them is possible in polynomial time. Combining the rapidly mixing Markov chain and uniform sampler from the sets provides an FPAUS algorithm. Since the #DCJ problem is self-reducible, it also gives an FPRAS algorithm.

A simpler Markov chain directly converging to the uniform distribution of all DCJ scenarios is also possible. Braga and Stoye [26] proved that any DCJ scenario can be obtained from any other DCJ scenario by successive transformations where each transformation changes only two consecutive DCJ operations. Therefore a Markov chain that randomly changes two consecutive DCJ operations in a DCJ scenario explores the entire solution space and, using standard Metropolis-Hastings techniques [27,28], it will converge to the uniform distribution. Since this Markov chain uses small perturbations, it is easy to see that the chain has a small diameter (*O*(*n*^2^) perturbations is sufficient to get from any DCJ scenario to any other), this chain is also a candidate for rapid mixing and thus for an FPAUS algorithm. However, giving a formal proof of rapid mixing of this chain seems to be surprisingly hard and is still a remaining problem to be solved.

Ouangraoua and Bergeron [29] and Braga and Stoye [26] gave polynomial algorithms to count the number of DCJ scenarios for co-tailed genomes or when the number of even length paths in the adjacency graph is limited. However, when the number of even length paths in the adjacency graph is not bounded, there is no fast algorithm to count the number of DCJ scenarios, and thus #DCJ is conjectured to be #P-complete.

#### The SCJ model

The Single Cut or Join model is computationally the simplest genome rearrangement model [21]. The decision/optimization counterpart of all the listed five problems are in P, therefore computational intractability of the counting versions cannot be directly concluded from the complexity of decision/optimization problems. Some of the counting problems under the SCJ model are easy (are in FP), some of them are computationally intractable (are in #P-complete and have no FPRAS approximations assuming that RP ≠ NP), and some of them have unknown computational complexity as described below.

Counting the number of most parsimonious SCJ operations is in FP as proved in [30]. Feijão and Meidanis [21] proved that there is a unique optimal SCJ median for 3 genomes. Their proof trivially extends to show that the optimal median remains unique for an arbitrary odd number of genomes: the optimal median contains the set of adjacencies that can be found in the majority of the genomes. Indeed, the SCJ distance between two genomes *G*_1 _and *G*_2 _is simply |Π_1_ΔΠ_2_|, where Π*_i _*is the set of adjacencies in *G_i_*. The key observation is that it is impossible that two conflicting adjacencies are present in more than half of the genomes, therefore the genome that contains exactly the adjacencies that are present in the majority of the genomes is a valid genome.

When the number of genomes is even, each extremity is in at most two adjacencies that are present in exactly half of the genomes. It is easy to see that an optimal median contains the set of adjacencies that are present in more than half of the genomes and any conflict-free subset of the adjacencies that are present in exactly half of the genomes. The number of optimal medians can be counted in the following way.

Given a set of genomes $G=\left\{G_{\text{1}},\;G_{\text{2}},...G_{\text{2}k}\right\}$ having the same synteny blocks, we define the conflict graph *C*(*V, E*) in the following way: The vertex set *V *is the set of extremities present in G  and there is an edge between *v*_1 _and *v*_2 _if and only if the adjacency (*v*_1_, *v*_2_) is present in exactly half of the genomes.

**Observation 1 ***The maximum degree of any vertex in C is 2*.

*Proof *This follows from the fact that any extremity can be in at most two adjacencies which are present in exactly half of the genomes.

The consequence of Observation 1 is that *C *can be decomposed into isolated vertices, paths and cycles. Any conflict-free subset of the adjacencies is a matching (non-necessary maximum and possibly empty) of *C*. The number of matchings is the product of the number of matchings on each component. Therefore it suffices to count this number. It is well-known [31] that the number of matchings in a length *n *path is

$$\overset{\left\lfloor \frac{n}{2}\right\rfloor }{\underset{k=1}{\sum }}\left(\begin{array}{c} n-k\\ k \end{array}\right)$$

and the number of matchings in a length *n *cycle is

$$\overset{\left\lfloor \frac{n}{2}\right\rfloor }{\underset{k=1}{\sum }}\frac{n}{n-k}\left(\begin{array}{c} n-k\\ k \end{array}\right).$$

Since obtaining the conflict graph, decomposing it into paths and cycles, counting the number of matchings on each component and multiplying these numbers all can be done in polynomial time, we can announce the following theorem:

**Theorem 1 ***The number of optimal medians under the SCJ model is in FP*.

Although calculating the number of optimal medians is easy, recently Miklós and Smith [32] proved that the number of most parsimonious median scenarios is in #P-complete. The proof uses a technique (modulo prime number calculations) that is typically used in those #P-complete problems that admit an FPRAS approximation. Define a simple Markov chain that walks on the optimal median genomes by adding or removing a random adjacency and converges to the distribution proportional to the number of scenarios that the median genome has by applying the Metropolis-Hastings algorithm [27,28]. Miklós and Smith proved that this Markov chain is torpidly mixing even if the number of genomes are fixed to 4, and only the size of the genomes are allowed to grow (unpublished result). Therefore it is absolutely unclear whether the number of most parsimonious median scenarios under the SCJ model has an FPRAS approximation or an FPRAS approximation would imply RP = NP. If the problem is in FPRAS, one will need a deeper understanding of the solution space to employ a more a sophisticated Markov chain method.

The number of most parsimonious scenarios on evolutionary trees under the SCJ model is known to be computationally intractable. Miklós and Smith [32] proved that it is #P-complete and Miklós, Tannier and Kiss [30] proved that it does not have an FPRAS approximation assuming RP ≠ NP. On the other hand, counting the number of most parsimonious labelings of evolutionary trees has an unknown computational complexity. One optimal labeling can be found by applying the Fitch algorithm [33] on each adjacency and choosing the absence of the adjacency at the root when the Fitch algorithm says that both the presence and absence of the adjacency give the minimum number of necessary SCJ mutations for that particular adjacency. Feijão and Meidanis [21] proved that the so-obtained genomes will always be valid. It is known that the Fitch algorithm cannot find all most parsimonious solutions for a particular character. The Sankoff-Rousseau algorithm [34] is a dynamic programming algorithm that is capable of finding all optimal solutions for a particular character, in the case of the SCJ model. However, it is easy to show that some solutions might be invalid, as conflicting adjacencies might be assigned to a genome labeling an internal node (making the genome and thus the solution invalid). Therefore, the solution space of optimal labelings is only a subset of the set that the Sankoff-Rousseau algorithm gives. It is known, when there is no constraint among the characters, the number of optimal labelings is in FP [35] and the number of most parsimonious scenarios is not in FPRAS assuming that RP ≠ NP [30]. Therefore the computational intractability of counting the number of most parsimonious scenarios on binary trees under the SCJ model by no means implies that counting the most parsimonious labelings would be a hard computational problem. On the other hand, the constraints among the adjacencies make the counting problem more complicated than the constraint-free version. It is unclear if this particular counting problem is in FP or #P-complete and, if it is in #P-complete, whether or not it has an FPRAS approximation.

In the next section we give a Gibbs sampler, exploring the solution space of the most parsimonious labelings, that seems to be rapidly mixing on some real life data. However, these examples can give only experimental evidence of rapid mixing suggesting that the problem might have an FPRAS approximation.

### Gibbs sampling of most parsimonious labeling of evolutionary trees under the SCJ model

Gibbs sampling is a special version of Markov chain Monte Carlo when the multivariate target distribution is hard to sample from, however, the conditional distribution of each variable is easy to sample [36]. This is exactly the case for the most parsimonious labelings of an volutionary tree under the SCJ model, as we show below.

#### Description of the Gibbs sampler

Let a rooted binary tree, *T *(*V, E*) be given, together with a function *f *mapping genomes under the SCJ model to the leaves of the tree, *L*. We assume that all genomes appearing as an image for some leaf have the same labels for their edges. Let A  represent the set of all adjacencies in ∪_*v*∈*L*_*f *(*v*). Let an arbitrary indexing on A  be given, then each genome *G *can be represented as a 0-1 vector **x **where *x_i _*is 1 if and only if *a_i _*∈ A  is in *G*. A length |A | 0-1 vector, **x**, is called valid if for all pairs of coordinates satisfying *x_i _*= *x_j _*= 1, adjacencies *a_i _*and *a_j _*do not share an extremity. Each valid vector represents a valid genome.

Genomes labeling the vertices of *T *are represented by such 0-1 vectors, and the Gibbs sampler works on these representations. The target distribution is the uniform distribution of the possible most parsimonious labelings. Consider any most parsimonious labeling as a set of vectors representing the genomes labeling the vertices of *T*. Choose one coordinate, *i*, then Gibbs sampling is to sample uniformly from all possible most parsimonious labelings that match the current labeling in all coordinates except coordinate *i*.

Formally, given a most parsimonious labeling of the internal nodes, a Gibbs sampling step is the following:

1 Draw a random coordinate *i *uniformly from 1, 2,...|A |.

2 Consider the *i*th coordinates of the vector representations of the genomes labeling the leaves, and on these 0-1 characters, do the Sankoff-Rousseau dynamic programming algorithm. For each leaf *l*, assign the value *s*(*l, k*) = 0 if *k *is the character assigned to *l *and *s*(*l, k*) = ∞ otherwise.

For each vertex *v *with children *u*_1 _and *u*_2_, the recursion is

(4) $$\begin{array}{ccc} s\left(v,0\right) & = & \text{min}\left\{s\left(u_{1},0\right),s\left(u_{1},1\right)+1\right\}+\\ & & \text{min}\left\{s\left(u_{2},0\right),s\left(u_{2},1\right)+1\right\} \end{array}$$

(5) $$\begin{array}{ccc} s\left(v,1\right) & = & \text{min}\left\{s\left(u_{1},0\right)+1,s\left(u_{1},1\right)\right\}+\\ & & \text{min}\left\{s\left(u_{2},0\right)+1,s\left(u_{2},1\right)\right\} \end{array}$$

**Figure 1:**
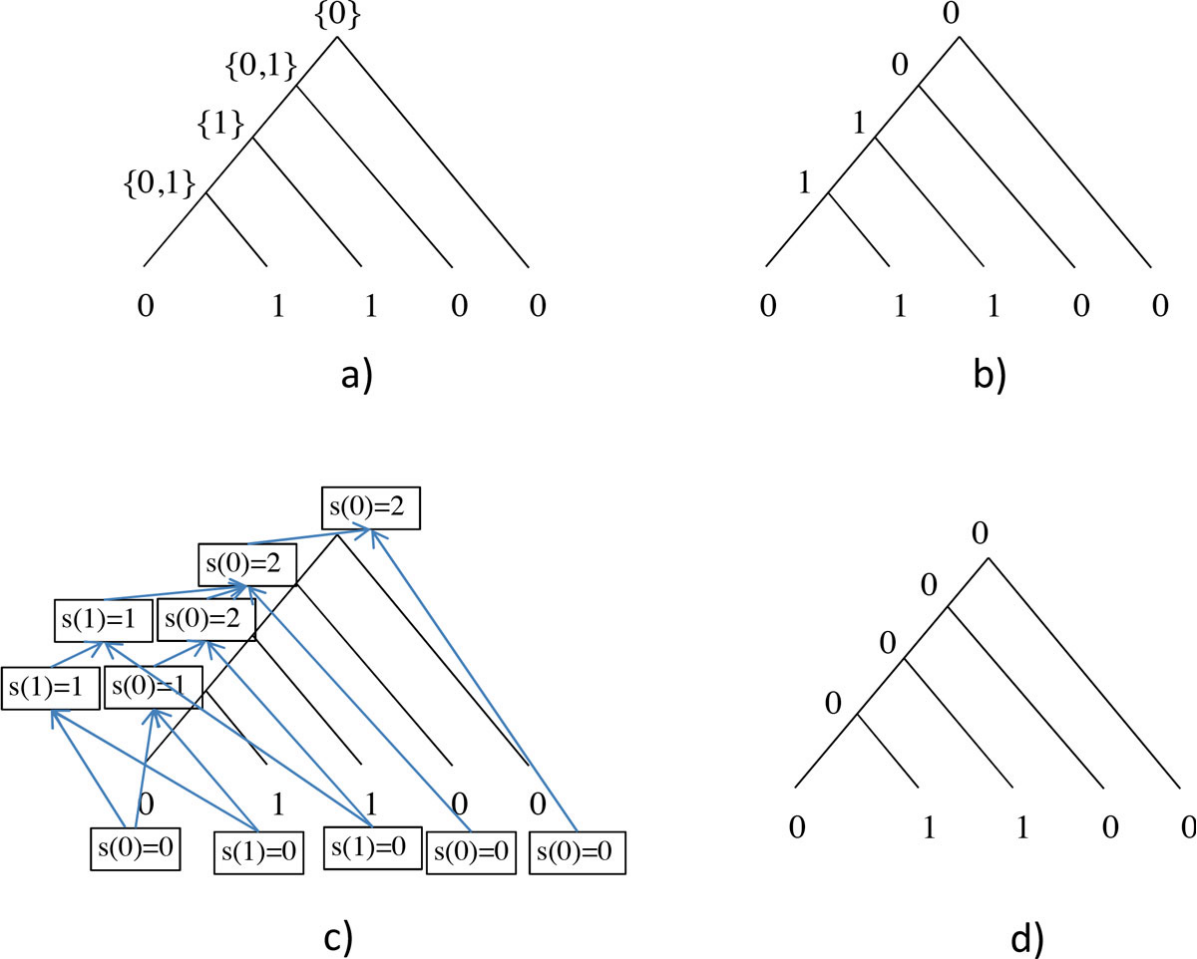
**A rooted binary tree with two most parsimonious labelings of internal nodes**. **a) **The *B *functions of the Fitch algorithm calculated in the bottom-up phase. **b) **The (canonical) Fitch solution. **c) **The values calculated in the Sankoff-Rousseau algorithm and the edges in the metagraph *M *(see text for details). For readability, only those values are indicated that contribute in estimating the number of most parsimonious solutions. Also, vertices of the tree are not indicated, i.e. *s*(*k*) is written instead of *s*(*v, k*). From positioning, it should be obvious which *s *value belongs to which vertex. **d) **The most parsimonious solution that can be obtained only by the Sankoff-Rousseau algorithm and not by the Fitch algorithm.

3 Create a directed metagraph *M *whose vertices are *s*(*v*, 0) for each vertex *v *of the tree and also those *s*(*v*, 1) for which writing 1 into the *i*th coordinate of the vector representing the genome labeling vertex *v *still a valid vector. Draw a directed edge from *s*(*u, k*) to *s*(*v*, *k'*) if *s*(*u, k*) gives the minimum for *s*(*v*, *k'*) in Equations (4) and (5). See also Figure 1c) and 1d).

4 Do an enumeration dynamic programming on *M*. Let *m*(*w*) = 1 if *w *= *s*(*l*, *k*), *k *∈ {0, 1} and *l *is a leaf. For other nodes, do the following. Let *w *= *s*(*v*, *k*), *k *∈ {0, 1}, and let the two children of *v *in the tree *T *be *u*_1 _and *u*_2_. Let $U_{1}$ denote the set of in-neighbors of *w *that are of the form *s*(*u*_1_, *k*) for *k *∈ {0, 1} and $U_{2}$ denote the set of in-neighbors of *w *that are of the form *s*(*u*_2_, *k*) for *k *∈ {0, 1}. Then

(6) $$m\left(w\right)=\left(\underset{z_{1}\in U_{1}}{\sum }m\left(z_{1}\right)\right)\cdot \left(\underset{z_{2}\in U_{2}}{\sum }m\left(z_{2}\right)\right)$$

where *m*(*w*) is called the weight of *w*.

5 If there is only one vertex in the metagraph *M *that is *s*(*root, k*), *k *∈ {0, 1}, choose that one at the root. Otherwise, choose randomly from the two vertices following the distribution proportional to their weights. For the chosen vertex *w*, *m*(*w*) is not 0, therefore it has at least 1 in-neighbor from both $U_{1}$ and $U_{2}$. From both in-neighbor sets, choose a random vertex from the distribution proportional to their weight, or the only one if only one vertex is in a set. Propagate down this process along the tree, thus one vertex from *M *is selected for each vertex of the tree *T*. Update the *i*th coordinates of the vectors according to the selected metagraph vertices: if *w *= *s*(*v, k*) was selected for vertex *v *then write *k *into the *i*th coordinate of the vector representing the genome labeling vertex *v*.

It is well-known that the number of most parsimonious labelings by one character can be calculated by Equation (6) [35], and when some of the solutions should be excluded due to some constraints, they simply should be omitted from the calculations. This is how the metagraph *M *was constructed. It is also a folklore that following the distribution proportional to the weights calculated in a recursion leads to the uniform distribution over the cases that the recursion calculates, and the uniform distribution is the one that we would like to sample from in the Gibbs sampling.

#### Irreducibility of the Gibbs sampler

The Gibbs sampler, as a Markov chain, will converge to the prescribed distribution if the Markov chain is irreducible, that is, any most parsimonious labeling can be transformed into any another by a finite number of Gibbs sampling steps. Due to the constraints on the coordinates, it is not trivial. Below we prove irreducibility by proving that any most parsimonious labeling can be transformed to a canonical labeling, the one described by Feijão and Meidanis [21]. Below we formally define this most parsimonious labeling. First, we recall the Fitch algorithm.

**Definition 6 ***The Fitch algorithm *[33]*is a greedy algorithm for finding a most parsimonious labeling of a tree, given a rooted binary tree, and the leaves of the tree are labeled by characters from some finite set. It has two phases (see also *Figure 1a)*and *1b)*)*.

*1 (Bottom-up phase) For each leaf v, assign a set B*(*v*) = {*c*} *where c labels v. Then for each internal node v with children u*_1 _*and u*_2_

(7) $$B\left(v\right)=\left\{\begin{array}{c} B\left(u_{1}\right)\cap B\left(u_{2}\right),\;if\;B\left(u_{1}\right)\cap B\left(u_{2}\right)\;is\;not\;empty\\ B\left(u_{1}\right)\cup B\left(u_{2}\right),\;otherwise. \end{array}\right\}$$

*2 (Top-down phase) Choose any member from B*(*root*) *to label the root. This is denoted by F *(*root*). *Then propagate down characters labeling internal nodes on the tree using the following recursion, where v is the parent of u*,

(8) $$F\left(u\right)=\left\{\begin{array}{cc} F\left(v\right)\cap B\left(u\right), & if\;F\left(v\right)\cap B\left(u\right)\;is\;not\;empty\\ any\;member\;from\;B\left(u\right), & otherwise. \end{array}\right\}$$

Although Equation (8) might be ambiguous for alphabets with size larger than 2, for 0-1 alphabet, there is no ambiguity. Ambiguity for 0-1 alphabet can happen only at the root when *B*(*root*) = {0, 1}.

**Definition 7 ***Let T *(*V*, *E*) *be a rooted binary tree with genomes labeling the leaves of the tree. Assume that each genome is represented as a 0-1 vector indicating which adjacency can be found in the genome, as described above. Then the canonical solution for the most parsimonious labeling of the tree under the SCJ model is given by applying the Fitch algorithm for each position of the representing vectors, and choosing 0 at the root whenever B*(*root*) = {0, 1}. *The so-obtained values are the coordinates of the vectors representing the genomes labeling the internal nodes of the tree*.

Feijão and Meidanis proved that the so-obtained vectors are always valid, thus they indeed give a most parsimonious labeling of the internal nodes [21]. Below we show that any solution to the most parsimonious labeling of the internal nodes under the SCJ model (which might be a solution that cannot be obtained by the Fitch algorithm just by the Sankoff-Rousseau algorithm, see for example, Figure 1d)) can be transformed into the canonical solution by a finite series of Gibbs sampling steps. First we have to prove a lemma regarding the values calculated in the Fitch algorithm and the Sankoff-Rousseau algorithm.

**Lemma 1 ***Assume T is an arbitrary rooted binary tree with leaves labeled by 0s and 1s. Then for any internal node v, B*(*v*) = {0, 1} *if and only if s*(*v*, 0) = *s*(*v*, 1).

*Proof *The ⇒ direction was proved in [30]. The ⇐ direction is proved by strong induction on *h*, the height of *v*. We prove the equivalent form *B*(*v*) ≠ {0, 1} ⇒ *s*(*v*, 0) ≠ *s*(*v*, 1). When *h *= 0, *v *is a leaf, and the statement is true as *s*(*v*, 0) ≠ *s*(*v*, 1) and *B*(*v*) ≠ {0, 1}.

For any node *h *≥ 1, assume that the statement holds for any node with height *k *<*h*. If *B*(*v*) ≠ {0, 1} then either *B*(*v*) = {0} or *B*(*v*) = {1}. The two cases are symmetric, so we might assume that *B*(*v*) = {0}, the proof for the other case is symmetric.

If *B*(*v*) = {0} and *u*_1 _and *u*_2 _are the children of *v*, then either *B*(*u*_1_) = *B*(*u*_2_) = {0} or *B*(*u*_1_) = {0}, *B*(*u*_2_) = {0, 1} or *B*(*u*_1_) = {0, 1}, *B*(*u*_2_) = {0}.

If *B*(*u*_1_) = *B*(*u*_2_) = {0}, then by the induction, *s*(*u*_1_, 0) ≠ *s*(*u*_1_, 1), and since the Fitch algorithm gives a most parsimonious solution, *s*(*u*_1_, 0) <*s*(*u*_1_, 1). Similarly for the other node, *s*(*u*_2_, 0) <*s*(*u*_2_, 1). Then *s*(*v*, 0) <*s*(*v*, 1), according to Equations (4) and (5).

If for one of the children, the *B *function takes {0, 1}, then for that node *u*, *s*(*u*, 0) = *s*(*u*, 1). For the sibling node *u'*, *s*(*u'*, 0) <*s*(*u'*, 1), and it is easy to check (by considering Equations (4) and (5)) that *s*(*v*, 0) <*s*(*v*, 1).   □

**Lemma 2 ***Let L  be a most parsimonious labeling of a tree T *(*V, E*) *under the SCJ model. Assume that the genomes are given in a binary vector representation as described above. Let v be the minimum height node for which some adjacency α, B_α_*(*v*) = {0}, *however, α is present in the genome labeling v (B_α_*(*v*) *is the set that the Fitch algorithm calculates for the vertex v when the algorithm is applied to the presence/absence of adjacency α). Change the current labeling in the following way. Remove α from the genome labeling the node v and propagate down the presence-absence of adjacency α below the subtree rooted in v according to the Fitch algorithm as v was the root of the tree. Then the so obtained new labeling *$L^{\prime }$

**a) ***contains valid genomes and*

**b) ***is also a most parsimonious labeling*.

*Proof *Changing any presence to absence cannot turn a valid genome into invalid. The only case when the genome might become invalid is when an absence is turned into presence (a possible example for this is on Figure 1d) and 1b), **d) **is a Sankoff-Rousseau solution, **b) **is the canonical Fitch solution). This might be the case when

• for some node *u *below *v*, *B_α_*(*u*) = {1} or

• on connected parts *C *of the tree where for all nodes, *u *∈ *C*, *B_α_*(*u*) = {0, 1}, except for the root of *C*, *r*, for which *B_α_*(*r*) = {1}.

If *B_α_*(*u*) = {1} then for all adjacencies *β *being in conflict with *α*, *B_β _*(*u*) = {0} (Lemma 6.1. in [21]). But then *β *must be absent in the genome labeling *u *otherwise it would contradict the minimum height of *v*.

For any connected part *C *with the above described property, we prove that for any adjacency *β*, which is in conflict with *α*, *β *is absent in the genomes labeling the vertices of *C*. For the root *r*, it holds as *B_α_*(*u*) = {1}, thus *B_β _*(*u*) = {0}. For any node *u *∈ *C*, for whose parent *w*, we showed that *β *is absent in the genome labeling *w*, we show that *β *is also absent in the genome labeling *u*. If *B_β _*(*u*) = {0}, then *β *is absent in the genome labeling *u *due to the minimal height of *v*. If *B_β _*= {0, 1}, then *s_β _*(*u*, 0) = *s_β _*(*u*, 1). Then in a most parsimonious labeling, it cannot be the case that *β *is absent in the genome labeling *w *but is presented in the genome labeling *u*. Indeed, such a labeling would have a parsimony score 1 for the edge (*u, v*), and a cost *s_β _*(*u*, 1) below the subtree rooted in *u*. On the other hand, if we change the labeling at the node *u *that *β *is absent in the genome labeling *u*, and on the subtree below *u*, we can change the presence/absence of *β *to get a parsimony score *s_β _*(*u*, 0). Then the parsimony score regarding *β *for the edge (*u*, *v*) is 0, hence this new labeling has a smaller total cost on the tree compared to the current one, a contradiction. By induction, on the whole connected part *C*, *β *is absent in the genomes labeling the vertices of *C*.

We proved that the new labeling $L^{\prime }$ contains valid genomes. We are going to prove that it is also a most parsimonious labeling. Since *B_α_*(*v*) = {0}, it follows that *s_α_*(*v*, 0) <*s_α_*(*u*, 1). Hence, in the old labeling L , the parsimony score regarding *α *on the subtree rooted in *v *was greater than in the modified labeling. On the edge connecting *v *to its parent, the new score might be 1, the old score might be 0, and then here the parsimony score might increase by 1, however, this loss cannot be greater than the gain we obtained on the subtree rooted at *v*. (And if the old labeling L  was most parsimonious it turns out that the old parsimony score regarding *α *on edge connecting *v *to its parent was 0.)

Since the number of adjacencies as well as the height of the tree is finite, in a finite number of steps, any labeling can be transformed into a labeling such that for all vertices *v *and all adjacencies *α*, *B_α_*(*v*) = {0} indicates that adjacency *α *is absent in the genome labeling *v*. Next, we consider transforming such labelings.

**Lemma 3 ***Let L  be a most parsimonious labeling of a tree T *(*V, E*) *under the SCJ model. Assume that the genomes are given in a binary vector representation as described above. Furthermore, assume that for all vertices w and all adjacencies α, B_α_*(*w*) = {0} *indicates that adjacency α is absent in the genome labeling w*.

*Let v be the minimum height node for which there is an adjacency α with B_α_*(*v*) = {1}, *however α is absent in the genome labeling v. Change the current labeling in the following way. Add α to the genome labeling the node v and propagate down the presence-absence of adjacency α below the subtree rooted in v according to the Fitch algorithm as v was the root of the tree. Then the so obtained new labeling *$L^{\prime }$

**a) ***contains valid genomes and*

**b) ***is also a most parsimonious labeling*.

*Proof *The proof of validity in Lemma 3 is exactly the same as the proof of Lemma 2 with one replacement. Each argument that said "if *B_β _*(*u*) = {0}, then *β *is absent in the genome labeling *u *due to the minimal height of *v*" should be replaced with "if *B_β _*(*u*) = {0}, then *β *is absent in the genome labeling *u *due to the given conditions."

Proving that the new labeling $L^{\prime }$ is also most parsimonious is exactly the same as the proof of Lemma 2, just switching 0 and 1.

Hence any most parsimonious labeling can be transformed to a most parsimonious labeling such that for each node *v *and each adjacency *α*, *B_α_*(*v*) = {0} indicates the absence of *α *in the genome labeling *v*, and *B_α_*(*v*) = {1} indicates the presence of *α *in the genome labeling *v*. Furthermore, each transformation is a possible Gibbs sampling step since one coordinate is changed from a most parsimonious labeling to another most parsimonious, valid labeling. During these transformations, when the labeling is changed below a vertex *v*, for which *B_α_*(*v*) ≠ {0, 1} for some *α*, the new labeling is the canonical Fitch labeling. What about the subtrees below the vertices *v *and the adjacencies *α *for which *B_α_*(*v*) = {0} where the adjacency *α *was absent in the initial labeling or *B_α_*(*v*) = {1} where the adjacency *α *was present in the initial labeling? The following lemma claims that, for such subtrees, the initial labeling was already the Fitch labeling.

**Lemma 4 ***Assume that in a most parsimonious labeling, B_α_*(*u*) = {0, 1} *and α is present (respectively, absent) in the genome labeling the parent of u. Then α is present (respectively, absent) in the genome labeling u*.

*Proof *Assume that the presence/absence of *α *in *u *and its parent is different. Then the parsimony score on the edge connecting *u *to its parent is 1. However, *s_α_*(*u*, 0) = *s_α_*(*u*, 1), hence switching the presence/absence of *α *is possible without changing the parsimony score on the subtree rooted at *u *(changing the presence/absence of *α *in genomes labeling vertices below *u *might be needed). On the other hand, the parsimony score on the edge connecting *u *to its neighbor could decrease by 1, a contradiction to the assumption that we started with a most parsimonious labeling.

The consequence of the Lemma 4 is that we can transform, by finite series of Gibbs sampling steps, any most parsimonious labeling to a labeling $L^{\prime }$ such that for all vertices *u *and all adjacencies *α*, for which *B_α_*(*u*) ≠ {0, 1} or a vertex *v *above *u *(*v *is not necessarily the parent of *u*, but may be an arbitrary node which is higher, but still above, *u*) exists such that *B_α_*(*v*) ≠ {0, 1}, the genome labeling *u *is the Fitch canonical solution regarding adjacency *α*. These labelings are almost in the Fitch canonical solutions, except for connected parts *C *containing the root of the tree on which for some *α*, *B_α_*(*v*) = {0, 1}, ∀*v *∈ *C*. The next lemma claims that they can be transformed into the Fitch canonical solution.

**Lemma 5 ***Let *L *be a most parsimonious labeling of a tree T *(*V*, *E*) *under the SCJ model. Assume that the genomes are given in a binary vector representation as described above. Furthermore, assume that for all vertices w and all adjacencies α, B_α_*(*w*) = {0} *indicates that adjacency α is absent in the genome labeling w and B_α_*(*w*) = {1} *indicates that the adjacency is present in the genome*.

*Consider any adjacency α, and let C denote the connected subset C containing the root for which B_α_*(*v*) = {0, 1}, ∀*v *∈ *C. (C might be the empty set.) Change the current labeling L  such that in the new labeling $L^{\prime }$ adjacency α be absent in each genome labeling any vertex v *∈ *C, and do not change the labeling otherwise. Then the new labeling*

**a) ***is a valid labeling and*

**b) ***is also a most parsimonious labeling*

*Proof *Changing the presence to absence cannot make an invalid genome, therefore proving the validity is trivial.

For any vertex *v*, *B*(*v*) = {0, 1}, either the *B *function for both children is also {0, 1} or for one of the children it is {1} and for the other child it is {0}. Extend *C *to *C' *such that we add to *C *all the cherry motifs (a pair of children) for which the *B_α _*function is {1} for one of the children and {0} for the other child. We know from the condition that *α *is present in the genome labeling one of the children and is absent in the genome labeling the other child. If we do not change the current labeling at the leaves of *C'*, there are two possible most parsimonious labelings regarding adjacency *α*: i) *α *is presented in all genomes labeling the internal nodes, ii) *α *is absent in all genomes labeling the internal nodes. The latter is what $L^{\prime }$ contains.

We are ready to prove the main lemma.

**Lemma 6 ***Let *L *be a most parsimonious labeling of a tree T *(*V, E*) *under the SCJ model. Then *L *can be transformed into the canonical Fitch solution by finite series of Gibbs sampling steps*.

*Proof *In the first phase, while there is a vertex *v *and adjacency *α *such that *B_α_*(*v*) = {0} and *α *is present in the genome labeling vertex *v*, find the *α *and *v *with the minimal height and do the Gibbs sampling indicated in Lemma 2.

After the first phase, in the second phase, while there is a vertex *v *and adjacency *α *such that *B_α_*(*v*) = {1}, however, *α *is absent in the genome labeling *v*, find the *α *and *v *with the minimal height, and do the Gibbs sampling indicated in Lemma 3.

After the second phase, in the third phase, while there is an adjacency *α*, for which we have a nonempty connected part *C *containing the root with the property that ∀*v *∈ *C*, *B_α_*(*v*) = {0, 1} and *α *is present in any of the genomes labeling any of the vertices *v *∈ *C*, choose one of these adjacencies, and remove it from all genomes labeling the vertices in *C*. Since it yields a most parsimonious labeling, it is also a Gibbs sampling step.

After the third phase, the labeling is the Fitch canonical labeling.

The main lemma directly leads to the following theorem.

**Theorem 2 ***Any most parsimonious labeling of a tree under the SCJ model can be transformed into any another most parsimonious labeling by finite series of Gibbs sampling steps*.

*Proof *A most parsimonious labeling $L_{1}$ can be transformed into the canonical labeling $L_{c}$ and also labeling $L_{2}$ can be transformed into $L_{c}$ by Gibbs sampling steps. Note that the inverse of a Gibbs sampling step is also a Gibbs sampling step, thus $L_{1}$ can be transformed into $L_{2}$ by first transforming $L_{1}$ into $L_{c}$ then transforming $L_{c}$ into $L_{2}$ by the inverse transformation that moves $L_{2}$ into $L_{c}$.

#### Testing the Gibbs sampler on real life data

The Gibbs sampler method has been implemented in Java programming language, downloadable from http://www.renyi.hu/~miklosi/SCJ-Gibbs/. The genomes of 8 vertebrate species were used to test the Gibbs sampler: *Gallus gallus, Monodelphis domestica, Bos taurus, Canis familiaris, Rattus norvegicus, Mus musculus, Homo sapiens, Rhesus macaque*. Synteny blocks were obtained as described in [37]. Only those synteny blocks were kept that could be found in all the 8 species. The tree topology applied was in agreement with the tree topology in [37]. The initial most parsimonious labeling was obtained using the Fitch algorithm. 10^7 ^Markov chain steps (Gibbs sampling steps) were applied, samples were collected after each 10000 steps. No burn-in phase was applied as the aim was the investigation of the mixing of the Markov chain and not calculating any statistics from the samples.

**Figure 2:**
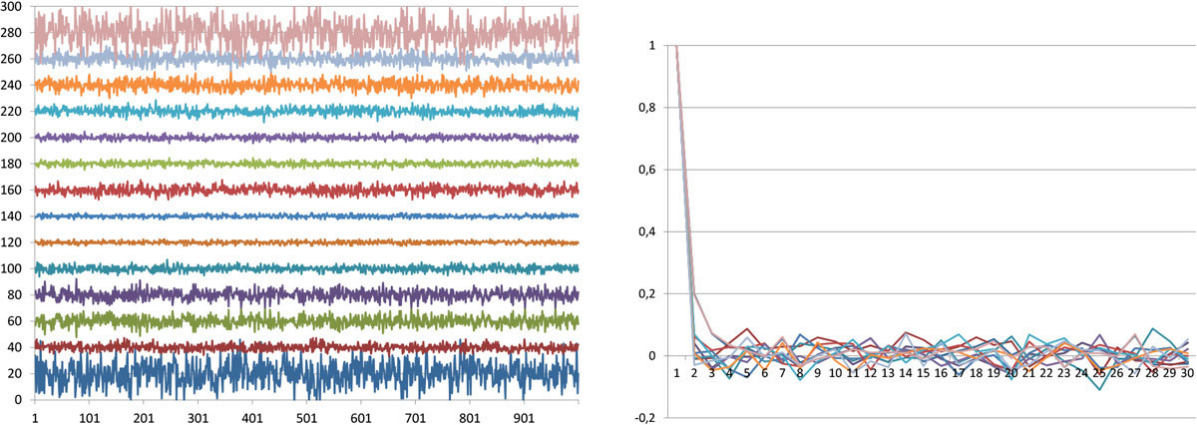
**Inferring the performance of the Gibbs sampler on 8 Vertebrates genomes**. See the text for detailed description of the data and the method. Left: The number of SCJ operations of the 14 edges of the evolutionary tree in the samples of the Gibbs sampler. Samples were collected after each 10000 Gibbs sampling steps, 1000 samples were collected. For readability, the numbers of SCJ operations falling onto edges have been shifted such that the average of them be 20, 40, 60,... 280. Right: Autocorrelations of the number of SCJ operations on edges in the samples. One unit on the first axis means 10000 Gibbs sampling steps.

Each sampled most parsimonious labeling has the same sum of edge lengths (number of mutations on an edge), however, the individual edge lengths vary during the Monte Carlo simulation. These lengths were used as traces of the Markov chain to empirically check the mixing of the Markov chain, see Figure 2. Note that the target distribution is the uniform distribution, thus the usual log-likelihood trace would be a constant line, and therefore, it could not be used for convergence analysis of the chain. As can be seen, the traces suggest good mixing: burn-in phase cannot be recognized on the trace plot. Autocorrelations are another statistics that can measure the mixing of Markov chains. 0 autocorrelation between samples at time *t *and *t *+ *k *means that *k *number of steps in the Markov chain is sufficient to get uncorrelated samples. The autocorrelations quickly approach to 0, also suggesting good mixing of the Markov chain.

## Discussion and conclusions

In this paper, we overviewed the state-of-the-art knowledge on the computational complexity of counting and sampling genome rearrangement scenarios. Most of the counting problems fall into one of the following three categories: i) easy to compute, i.e., the number of solutions can be exactly calculated in polynomial time, ii) hard to count exactly in polynomial time, however, stochastic approximations exist that are just as good in practice than exact calculations iii) hard to count both exactly and approximately.

Unfortunately, all counting problems whose decision/optimization counterparts are NP-hard fall into the third category. Surprisingly, counting the SCJ scenarios on a phylogenetic tree also falls into the third category, although its optimization counterpart is in P. Counting the number of SCJ scenarios between two genomes as well as counting the number of most parsimonious medians under the SCJ model is easy. Counting the number of most parsimonious DCJ scenarios has a good stochastic approximation. That approximation is given via a rapidly mixing Markov chain. This is a general phenomenon that sampling and counting have the same computational complexity and a solution to one of the problems explicitly gives a solution to the other problem. In applications, sampling is usually more important than counting, however, theoretical results on the computational complexity on counting naturally tells the limit of possibilities of sampling algorithms.

The most important open questions are:

• Is it possible to sample (almost) uniformly most parsimonious reversal scenarios between two genomes in polynomial time?

• Is it possible to sample (almost) uniformly most parsimonious SCJ median scenarios in polynomial time?

• Is it possible to sample (almost) uniformly most parsimonious labelings of an evolutionary tree under the SCJ model in polynomial time?

• Is it easy or hard to count exactly the most parsimonious labelings of an evolutionary tree under the SCJ model?

• Is it easy or hard to count exactly most parsimonious reversal scenarios?

• Is it easy or hard to count exactly most parsimonious DCJ scenarios?

The greatest effort has been made to develop a method efficiently sampling most parsimonious reversal scenarios between two genomes. Some Markov chain methods have been developed [24,9] that seem to work well in practice [38]. Unfortunately, no formal proof is given so far that these Markov chains are indeed rapidly mixing.

Above giving an overview of the computational complexity of counting genome rearrangement scenarios, we also gave a Gibbs sampler for sampling most parsimonious labelings of evolutionary trees under the SCJ model. Sampling and counting such labelings have unknown computational complexity. Our sampler works well in practice on real life data, and these experiments suggest the conjecture that at least good stochastic approximation exists for these problems. Although the SCJ model is one of the least realistic genome rearrangement models, there is a strong correlation between SCJ and DCJ distances. Therefore a rapidly mixing Markov chain on SCJ phylogenies could open the possibility to develop Monte Carlo methods for approximate DCJ phylogenies.

We considered only five special counting problems in this paper, each of them under three possible rearrangement models. There are further genome rearrangement problems like genome halving [39], guided genome halving [40], genome aliquoting [41]. Some of them are computationally easy as decision problems [42], therefore, it is a natural question what can we say about the computational complexity of counting the solutions of these problems.

## Competing interests

The authors declare that they have no competing interests.

## Authors' contributions

IM and HS considered the counting and sampling problems, set up the conjectures and proved the theorems. IM implemented the Gibbs sampler and tested it on real life data.
